# Analysis of the Epidemiological Trends on Inpatient Diverticulosis Admissions in the US: A Longitudinal Analysis From 1997–2018

**DOI:** 10.7759/cureus.34493

**Published:** 2023-02-01

**Authors:** Andre Fialho, Andrea Fialho, Asim Shuja

**Affiliations:** 1 Gastroenterology, Digestive Health Institute at Orlando Health, Orlando, USA; 2 Gastroenterology and Hepatology, University of Illinois Chicago, Chicago, USA

**Keywords:** health public, public health, hospital based, hospital, health care, diverticular disease of the colon

## Abstract

Background

Diverticulosis of the colon is characterized by outpouchings of mucosa and serosa through the muscular layer of the large intestinal wall. It is classically associated with increasing age with older individuals having a higher prevalence and greater density of diverticula secondary to its progressive disease nature. Also, diverticular disease is associated with dietary habits, low fiber intake in western society as well as obesity. The aim of this study was to investigate the epidemiological trends associated with diverticular disease in the United States in a 21-year interval from 1997 to 2018.

Methods

Using the Nationwide Inpatient Sample, all hospitalizations between 1997 and 2018 were analyzed. We examined annual data for hospitalization rate, the average length of stay (LOS), mean age and interval age groups, and hospital charges for inpatient admissions for diverticular disease (diverticulitis and diverticulosis).

Results

Between 1997 and 2018, the number of hospitalizations for patients with a primary discharge diagnosis of diverticular disease (diverticulosis and diverticulitis) increased 32% from 220,896 to 293,530 with 89.7 discharges per 100,000 persons in 2018 versus 81.0 discharges per 100,000 persons in 1997.

Overall, the average age of patients decreased from 67.55 ± 0.15 years in 1997 to 64.59 ±0.08 in 2018, [t-value (t) 12.56, degrees of freedom (df) 514424, 95% confidence interval (CI) 2.497-3.423, P<0.0001]. On further evaluation, the mean average age in males decreased from 63.16±0.21 years in 1997 to 61.31±0.12 years in 2018, (t 8.16, df 217981, 95% CI 1.404-2.295 P<0.0001), while in females it decreased from 70.53±0.14 years to 67.15±0.10 years, (t 20.13, df 296422, 95% CI 3.050-3.709 P<0.0001), in the same interval time.

While evaluating different subgroups of age in this time interval, the prevalence rate of diverticular disease diagnosis per 100,000 persons increased in the interval age between 18-44 years from 20.1 to 29.8, [relative risk (RR) 0.848, CI 95% 0.834-0.863, P< 0.0001) and 45-64 years from 107.1 to 125.3, (RR 0.761, CI 95% 0.754-0.769 P<0.0001) while it decreased in the interval age between 65-84 years from 357.6 to 259.7, (RR 1.211, CI 95% 1.206-1.226, P<0.0001) as well as > 85 years from 746.2 to 523.6, (RR 1.130, CI 95% 1.112-1.147, P<0.0001)

The length of stay (LOS) mean average in days decreased from 5.8 ± 0.04 days in 1997 to 4.4±0.021 days in 2018, (t 33.08 df 514424, 95%CI 1.316-1.483, P< 0.0001).

Hospital Inpatient National Statistics data over hospital mean charges, available from the period between 1997 to 2015, shows that the mean hospital charges in US dollars increased over 100%, from $19,735.17 in 1997 to $39,575 in 2015 (P<0.001) even after adjusting values to 2015 inflation.

Conclusion

There is an overall trend of decreased mean age of patients admitted with diverticular disease in the US over the past 21 years with a respective significant increased rate of disease in younger age groups. We postulate that these changes may be associated with poor dietary habits and obesity epidemics worsened in the last two decades in the US. In addition, despite the decreased length of stay over the same time period, the mean hospital charges more than double likely reflecting the increased access to expensive diagnostic methods such as computed tomography and colonoscopies.

## Introduction

Diverticulosis of the colon consists of the development of false diverticula with outpouchings of mucosa and serosa through the bowel muscular layer. Diverticulosis is usually detected incidentally in asymptomatic patients undergoing endoscopies or radiological examinations, such as computed tomography (CT) or magnetic resonance imaging (MRI).

Diverticulosis is a common gastrointestinal condition historically affecting up to two-thirds of the overall population by the ninth decade of life and constituting one of the most common findings on colonoscopy with an increase in prevalence with increasing age [1,2].

Diverticular disease is likely multifactorial and is still not completely understood. Several factors appear to contribute to its development such as colonic wall structure, colonic motility, genetics, fiber intake, vitamin D levels, obesity, and possibly lack of physical activity [3].

The great majority of patients with diverticulosis remain asymptomatic but approximately 25% of individuals can develop symptomatic diverticulosis with diverticulitis, segmental colitis associated with diverticulosis, and symptomatic uncomplicated diverticular disease defined as the presence of episodes of abdominal pain in the absence of acute diverticulitis attack [4,5].

Classically, the prevalence of diverticulosis is considered very low in individuals <40 years of age and this condition usually affects more individuals > 65 years of age. Complications of diverticular disease are usually related to diverticular bleeding and diverticulitis [6].

There is a lack of studies evaluating changes in mean patient age as well as admission rates in the US for the past two decades. Therefore, the aim of this study was to investigate the epidemiological trends associated with diverticular disease in the United States in a 21-year interval from 1997 to 2018.

## Materials and methods

Study design and data source

We conducted a cross-sectional analysis of data available from 1997-2018 using the National Inpatient Sample (NIS). NIS data provides hospital administrative data through the Healthcare Cost and Utilization Project (HCUP) which is sponsored by the Agency for Healthcare Research and Quality. The NIS database is the largest publicly available all-payer inpatient care database in the United States and contains data from more than eight million hospital stays every year. The NIS sampling frame covers > 95% of the United States population and 94% of all community hospital discharges. All NIS data were subject to a data use agreement. This study was exempt from Institutional Board Review.

Identification of diverticular disease hospitalizations

We identified all hospitalized patients, from age one year old and above, discharged with diverticulosis at the Hospital Inpatient National Statistics data using the clinical classification software refined (CCSR) with principal diagnosis: DIG013 (diverticulosis and diverticulitis) as the primary diagnosis. This includes all patients with a diagnosis of diverticulosis and its complications.

CCSR is one in a family of databases and software tools developed as part of the Health Cost and Utilization Project (HCUP) a Federal-State-Industry partnership sponsored by the Agency for Healthcare Research and Quality (AHRQ).

CCSR aggregates International Classification of Diseases, 10th Revision, Clinical Modification/Procedure Coding System (ICD-10-CM/PCS) codes into clinically meaningful categories.

The CCSR for ICD-10-CM diagnoses is intended to be used analytically to examine patterns of healthcare in terms of cost, utilization, and outcomes; rank utilization by diagnoses; and risk adjust by clinical condition. 

Variables collected

Patient demographics collected included age, sex, and the median income per zip code. Hospital characteristics collected included location (Northeast, Midwest, South, and West). Hospital charges are defined as the amount the hospital charges for the patient’s entire hospital stay, but it does not include professional (physician) fees. Length of stay is defined as the number of nights the patient remained in the hospital per stay.

Statistical analysis

Continuous variables were presented as mean ± standard deviation (SD) or N%. Student t-tests were used for continuous factors, and Pearson chi-square tests were used for categorical variables. Frequencies and percentages for categorical variables, means, and standard errors for numeric variables were reported. Rates of diverticular disease per 100,000 admissions were calculated by dividing the total number of patients with diverticular disease as primary discharge diagnosis by the total number of all discharges listed each year. Pearson chi-square test was used to compare proportions between 1997 and 2018. Relative risk ratios (RR) along with their 95% confidence intervals (CI) comparing 1997-2018 were estimated. Annual charges reported are the raw charges adjusted for the long-term average healthcare annual inflation rate (https://www.usinflationcalculator.com), so all dollar values are related to 2018 dollars. 

All analyses were done using MedCalc for Windows and GraphPad.

## Results

Between 1997 and 2018, the number of hospitalizations for patients with primary discharge diagnoses of diverticular disease (diverticulosis and diverticulitis) increased by 32% from 220,896 to 293,530. The rate of hospital discharges for diverticulosis increased to 89.7 discharges per 100,000 persons in 2018 versus 81.0 discharges per 100,000 persons in 1997 (P-<0.0001). (Table 1) 

**Table 1:** Number and rate of discharges with diverticular disease by patient’s characteristics in 1997 and 2018

Category	Categorical variable	1997	2018	1997	2018	1997	2018
		Diverticular disease	Diverticular disease	Per 100,000 admissions	Per 100,000 admissions	Age, mean (years)	Age, mean (years)
		(N, %)	(N, %)				
All discharges		220,896	293,530	81.0	89.7	67.55	64.59
Age group	1-17	85 (0.04)	60 (0.02)	0.1	0.1	11.98	14.0
	18-44	22,324 (10.11)	34,950 (11.91)	20.1	29.8	38.01	37.16
	45-64	60,261 (27.28)	105,095 (35.8)	107.1	125.3	55.15	55.46
	65-84	109,064 (49.37)	119,155 (40.59)	357.6	259.7	74.82	73.94
	85+	29,142 (13.19)	34,270 (11.68)	746.2	523.6	88.81	88.16
Sex	Male	89,363 (40.45)	128,620 (43.82)	67.0	79.8	63.16	61.31
	Female	131,519 (59.54)	164,905 (56.18)	94.5	99.3	70.53	67.15
Region	Northeast	47,737 (21.61)	59,405 (20.24)	90.5	105.9	66.79	65.10
	Midwest	49,310 (22.32)	66,805 (22.76)	77.8	97.8	67.70	64.73
	South	90,387 (40.92)	118,310 (40.31)	93.9	94.8	67.86	64.48
	West	33,463 (15.15)	49,010 (16.70)	55.5	62.8	67.58	64.06
Median income for zipcode	Low	69,172 (31.31)	75,115 (25.59)	94.4	*	69.18	64.32
	Not low	139,187 (63.01)	214,435 (73.05)	86.8	*	66.80	64.69

Overall, the average age of patients decreased from 67.55 ± 0.15 years in 1997 to 64.59 ±0.08 in 2018, [t-value (t) 12.56, degrees of freedom (df) 514424, 95% CI 2.497-3.423, P<0.0001]. On further evaluation, the mean average age in males decreased from 63.16±0.21 years in 1997 to 61.31±0.12 years in 2018, (t 8.16, df 217981, 95% CI 1.404-2.295 P<0.0001), and in females it decreased from 70.53±0.14 years to 67.15±0.10 years, (t 20.13, df296422, 95% CI 3.050-3.709 P<0.0001), in the same interval time. 

While evaluating different subgroups of age in this time interval, the prevalence rate of diverticular disease diagnosis per 100,000 persons increased in the interval age between 18-44 years from 20.1 to 29.8, (relative risk [RR] 0.848, CI 95% 0.834-0.863, P< 0.0001) and 45-64 years from 107.1 to 125.3, (RR 0.761, CI 95% 0.754-0.769 P<0.0001) while it decreased in the interval age between 65-84 years from 357.6 to 259.7, (RR 1.211, CI 95% 1.206-1.226, P<0.0001) as well as >85 years from 746.2 to 523.6, (RR 1.130, CI 95% 1.112-1.147, P<0.0001)

The average length of stay (LOS) in days decrease from 5.8 ± 0.04 days in 1997 to 4.4±0.021 days in 2018, (t 33.08 df 514424, 95%CI 1.316-1.483, P< 0.0001).

Additional data analysis showed a decrease in average age in all US regions more marked at the South 67.86± 0.26 to 64.48± 0.14 (t 12.15 df 208695, 95%CI 2.833-3.926, P< 0.0001) and West 67.58± 0.36 to 64.06± 0.22 (t 8.817 df 82471, 95%CI 2.735-4.304, P< 0.0001) Also, decrease average interval age on diagnosis was also significant in individuals with regardless of income based on zipcode (Table 1).

Despite the decrease in the average LOS, Hospital Inpatient National Statistics data over hospital mean charges, available from the period between 1997 to 2015, shows that the mean hospital charges in US dollars increased over 100%, from $19,735.17 in 1997 to $39,575 in 2015 (P<0.001) even after adjusting values to 2015 inflation.

## Discussion

Diverticulosis is a highly prevalent condition, affecting men and women equally, increasing significantly with aging and present in up to 50-60% of individuals above 80 years [6,7]. Diverticulosis complications are associated with significant inpatient admission and increased healthcare expenses, accounting for costs up to 2.1-2.6 billion dollars per year in the United States [8]. Diverticulosis complications are classically divided between diverticular bleeding and diverticulitis. Diverticular bleeding is caused by lesions to the intramural branches of the marginal artery at the dome or neck of the diverticulum and can cause severe and profuse bleeding. Mechanical and chemical trauma appears to play a major role in its development [7, 9]. Otherwise, diverticulitis, a peri-diverticular inflammation of the bowel wall and surrounding tissue is thought to be secondary to the translocation of intestinal bacteria through the mucosa of the diverticulum [10-12]. There have been recent changes in the classification of diverticulitis into different categories: chronic recurrent diverticulitis, segmental colitis associated with diverticulosis (SCAD), and symptomatic uncomplicated diverticular disease (SUDD). SUDD has been recently associated with Irritable Bowel Syndrome due to bowel hyperalgesia and dysmotility [13].

In this large national database study, we evaluated the demographics associated with diverticular disease in inpatient admissions in the US over a period of 21 years, from 1997 to 2018. Our results show that between 1997 and 2018 the total number of hospital discharges with a primary diagnosis of diverticular diseases increased by 32%.

Despite decreased LOS, mean hospital charges in US dollars doubled in the 21-year interval. Given the significant healthcare impact on utilization and costs associated with diverticulosis, it is important to understand and follow potential changes in disease rates and in patient profiles in order to determine strategies to mitigate disease complications and possibly increase clinician awareness of this condition.

Also, the mean age of diverticular disease admissions in the US significantly decreased with increased incidence of diagnosis in the age groups from 18-44 and 45-65-year-old as well as decreased rates in >65 years. These findings are interesting and may reflect an intergenerational decline in dietary habits and an increase in obesity rates now seen in younger generations, which are common risk factors for diverticulosis development. Increased access to diagnostic imaging tests and colonoscopy procedures may contribute to earlier detection of this condition but don’t fully explain lower rates in older subgroups noted on our interval evaluation.

Interestingly, US data from 2012 similarly encountered increased rates in groups aged 18-44 years and 45-65 years, in a smaller interval study performed with seven years data interval but no significant changes in disease rate were noticed in the group aged over 65 years [14]. These differences may be secondary to shorter data spam collected on the referred studies with additional year intervals included in our study being able to appropriately reflect declined rates in older age groups.

Although limited data is available so far, the presence of diverticulosis at younger ages appears to be associated with comorbidities such as an increase in visceral fat and may be associated with an increased risk of additional diseases including polyps and potentially colon cancer [15]. Coincidentally, a recent rise in colon cancer diagnoses in young individuals under age 50 with colorectal cancer has been widely reported [16]. Further studies are necessary to evaluate if similar risk factors are driving increased diverticulosis rates in younger patients noted in our study.

In summary, our study is important in highlighting the increased rate of diverticulosis in younger individuals in the US from 1997 to 2018. There is an overall trend of decreasing the mean age of patients admitted with a diverticular disease in the US over the past 21 years. Decreased LOS was not reflected in hospital charges and potential healthcare cost impacts increased. Further studies are necessary to understand the risk factors associated to increase diverticulosis rates in younger patients.

## Conclusions

There is an overall trend of decreased mean age of patients admitted with diverticular disease in the US over the past 21 years with a respective significant increased rate of disease in younger age groups. We postulate that these changes may be associated with poor dietary habits and obesity epidemics worsened in the last two decades in the US. In addition, despite the decreased length of stay over the same time period, the mean hospital charges more than double likely reflecting the increased access to expensive diagnostic methods such as computed tomography and colonoscopies.
